# miRNA Modulation and Antitumor Activity by the Extra-Virgin Olive Oil Polyphenol Oleacein in Human Melanoma Cells

**DOI:** 10.3389/fphar.2020.574317

**Published:** 2020-09-23

**Authors:** Sara Carpi, Beatrice Polini, Clementina Manera, Maria Digiacomo, Jasmine Esposito Salsano, Marco Macchia, Egeria Scoditti, Paola Nieri

**Affiliations:** ^1^Laboratory of Molecular Pharmacology, Department of Pharmacy, University of Pisa, Pisa, Italy; ^2^Interdepartmental Research Center “Nutraceuticals and Food for Health,” University of Pisa, Pisa, Italy; ^3^Laboratory of Medicinal Chemistry, Department of Pharmacy, University of Pisa, Pisa, Italy; ^4^Doctoral School in Life Sciences, University of Siena, Siena, Italy; ^5^Laboratory of Vascular Biology and Nutrigenomics, National Research Council (CNR) Institute of Clinical Physiology (IFC), Lecce, Italy

**Keywords:** melanoma, oleacein, extra-virgin olive oil, microRNA, mTOR, BCL2, apoptosis, polyphenols

## Abstract

Extra-virgin olive oil (EVOO) polyphenols contribute to Mediterranean diet health-promoting properties. One of the most abundant secoiridoid present in EVOO, Oleacein (OA), demonstrated anticancer activity against several tumors. Nevertheless, its role against melanoma has not still investigated. This study aimed at determining *in vitro* the antimelanoma activity of OA and the relative mechanism of action. OA induced cell growth inhibition in 501Mel melanoma cells with an IC50 in the low micromolar range of concentrations. Moreover, an OA concentration approximating the IC50 induced G1/S phase arrest, DNA fragmentation, and downregulation of genes encoding antiapoptotic (BCL2 and MCL1) and proproliferative (c-KIT, K-RAS, PIK3R3, mTOR) proteins, while increased transcription levels of the proapoptotic protein BAX. Concordantly, OA increased the levels of miR-193a-3p (targeting MCL1, c-KIT and K-RAS), miR-193a-5p (targeting PIK3R3 and mTOR), miR-34a-5p (targeting BCL2 and c-KIT) and miR-16-5p (miR-16-5p targeting BCL2, K-RAS and mTOR), while decreased miR-214-3p (targeting BAX). These modulatory effects might contribute to the inhibition of 501Mel melanoma cell growth observed after treatment with an olive leaves-derived formulation rich in OA, with potential application against *in situ* cutaneous melanoma. Altogether, these results demonstrate the ability of OA to contrast the proliferation of cutaneous melanoma cells through the transcriptional modulation of relevant genes and microRNAs, confirming the anticancer potential of EVOO and suggesting OA as a chemopreventive agent for cancer disease therapy.

## Introduction

The epidemiological observation that habitants of Mediterranean countries exhibit longevity and lower incidence of the age-related diseases has been closely linked to their food habits (Capurso et al., 2019). In this context, the consumption of extra-virgin olive oil (EVOO), obtained from the drupes of the olive tree (*Olea Europaea* L.), has been clinically associated with the health-promoting properties of the Mediterranean diet (Parkinson and Cicerale, 2016; Lozano-Castellón et al., 2019).

Compared to other vegetable oils, EVOO, in addition to high levels of oleic acid, is characterized by a high amount of phenolic and polyphenolic compounds endowed with antioxidant, antiinflammatory, vasculoprotective actions (Visioli and Bernardini, 2011), which have been reported to be responsible for EVOO main pharmacological and pharma-nutritional properties against the development of several chronic diseases, including cardiovascular diseases, obesity, diabetes, metabolic syndrome, and cancer (Visioli et al., 2018; Crespo et al., 2018). Among the nutraceutical properties, cancer prevention has been well recognized. Indeed, several epidemiological investigations have shown a lower incidence of tumors associated with regular olive oil consumption (Gerber, 1997; Visioli and Bernardini, 2011; Malagoli et al., 2019), with a contributory role by polyphenols whose chemopreventive activity is well documented and probably exerted through synergistic interactions (Visioli et al., 2020). Further studies that deepen the role of each polyphenol are needed to better understand their health-promoting contribution.

Cutaneous melanoma is a type of cancer with a greater increase in incidence in Western and Northern Europe (Dimitriou et al., 2018). Recently, a population-based case-control study in Northern Italy revealed an inverse correlation between melanoma risk and consumption of olive oil (Malagoli et al., 2019). Some single polyphenols have already been investigated in melanoma *in vitro* and *in vivo* models revealing their cytotoxic activity and anticarcinogenic action. Indeed, for instance, oleuropein, the main secoiridoid glucoside present in the *Olea europaea* leaves and also olive oil, induces the downregulation of the pAKT/pS6 pathway enhancing the cytotoxicity activity of different antimelanoma chemotherapeutic drugs (Ruzzolini et al., 2018). Hydroxytyrosol, the most representative simple phenol of EVOO and *Olea Europaea* L. leaves, causes inhibition of melanoma cell proliferation activating caspase-3-dependent apoptosis (D’Angelo et al., 2005).

As far as oleacein (3,4-(dihydroxyphenyl) ethanol (3,4-DHPEA-EDA)) (OA) is concerned, another abundant secoiridoid in EVOO and *Olea Europeae* L. tree (Castejón et al., 2020), no research has investigated so far its antimelanoma activity, despite its emerging antiproliferative activity in other cancer cells, i.e. cutaneous squamous cell carcinoma cells (a non-melanoma skin cancer) (Polini et al., 2018), multiple myeloma cells (Juli et al., 2019), promyelocutic leukemia cells (Fabiani et al., 2008) and very recently in neuroblastoma cells (Cirmi et al., 2020).

Against this background, the main aim of this study was the evaluation of the antimelanoma activity of OA in a cell model of cutaneous melanoma, and to investigate its mechanism(s) of action with particular attention to the effect on specific genes and microRNAs (miRNAs) involved in melanoma cell survival, proliferation and resistance to apoptosis. Specifically, we concentrated on the mammalian target of rapamycin (mTOR) signaling pathway, which is a positive regulator of cell growth and proliferation by promoting many anabolic processes (biosynthesis of proteins, lipids and organelles), and limiting catabolic processes, and is deregulated in human cancer (Saxton and Sabatini, 2017); and on the BCL2 family, which is overexpressed in many cancers including melanoma, and involves pro- and antiapoptotic proteins leading to the intrinsic or mitochondrial apoptotic response (Hata et al., 2015). We also tested the anticancer potential of an olive leaves-derived formulation for potential topic application, which is particularly rich in OA and its derivatives hydroxytyrosol and oleuropein with already known antimelanoma activities.

Our data showed an anticancer activity of OA against melanoma, demonstrating its ability to inhibit melanoma cell proliferation by regulating the expression of genes and related regulatory miRNAs involved in the mTOR pathway and the apoptotic process.

## Materials and Methods

### OA Extraction, Purification, and Characterization

The extraction of OA was performed using a previously reported procedure (Polini et al., 2018). For the purification of OA, we developed a more efficient method using advanced automated flash purification (Isolera™ Prime 3.2.2, Biotage®). As stationary phase, we chose a Biotage® SNAP Ultra cartridge (HP-Sphere™ 25µm), and as mobile phase, the mixture of CHCl_3_ (A) and ethyl acetate (B) shown in **Table 1**. The flow rate was 25 ml/min and for each tube 15 ml was collected.

**Table 1:** Chromatographic method.

Mobile Phase (B%)	Fractions	Volume (ml)
0%	1–36	540
From 0% to 45%	37–180	2,160
45%	181–186	90
From 45% to 100%	187–192	90
100%	193–198	90

Fractions 60–130 (about 60 mg) containing OA were subjected to a further purification by advanced automated flash purification (Isolera™ Prime 3.2.2, Biotage®) using a Biotage® SNAP Ultra C18 12g cartridge (HP-Sphere™ C18 25 µm) as stationary phase. The mobile phase was a mixture of H_2_O (A) and acetonitrile (B) shown in **Table 2**.

**Table 2:** Chromatographic method.

Mobile Phase (B%)	Fractions	Volume (ml)
From 5% to 10%	1–10	51
From 10% to 100%	11–61	255
100%	62–75	68

The flow rate was 10 ml/min and for each tubes 5 ml was collected.

Fractions 20–30 (about 10 mg) contained pure OA (purity > 95%). NMR and HPLC analysis confirmed the exact structure of OA.

### Reagents and Cell Culture

Human melanoma cells 501Mel (from melanoma metastasis) were kindly provided by Dr. Poliseno (Oncogenomics Unit, Core Research Laboratory, Istituto Toscano Tumori c/o IFC-CNR, Pisa, Italy). Cells were cultured in RPMI 1640 medium (Euroclone, Milan, Italy) supplemented with 10% fetal bovine serum (FBS), 100 U/ml penicillin, and 100 μg/ml streptomycin (Euroclone, Euroclone, Milan, Italy) in a humidified atmosphere containing 5% CO_2_ at 37°C. Cell morphology was examined under light microscopy. OA and Imiquimod (Merck Millipore, Darmstadt, Germany) were dissolved in DMSO and the glycerol concentrated extract of olive leaf polyphenols Oleacin^®^ (Arisi Giacomo e Figli s.r.l.) was dissolved in ethanol and water in ratio 9:3:2, as indicated in European Pharmacopoeia (Ph. Eur.).

### Cell Viability Assay

The ability of OA to interfere with melanoma cell growth was evaluated in 501Mel cells in a concentration range of 0.1–200 μM for 48 and 72 h. Cell proliferation was measured using the MTS tetrazolium compound (CellTiter 96 Aqueous One Solution Cell Proliferation assay; Promega, Madison, WI), following the manufacturer’s instructions. Briefly, cells (5 × 10^3^/well) were seeded onto 96-well plates and incubated with various concentrations of compounds for 48 and 72 h in 1% FBS-added medium, to avoid serum proteins interference with compounds. For each treatment, the corresponding vehicle-treated cells were used as control (Ctrl). Final concentration of each solvent in culture medium didn’t exceed 0.2%.

At the end of the treatment, 20 μl MTS solution was added to each well. Absorbance was read at 490 nm using Infinite M200 NanoQuant instrument (Tecan, Salzburg, Austria). Optical density values from vehicle-treated cells were considered as 100% cell viability and the drug concentration giving 50% cell growth inhibition (IC_50_) was calculated using GraphPad software (GraphPad Prism, version 8.0 from GraphPad Software Inc., San Diego, CA, USA).

### Cell Cycle Assay

The phosphorylation levels of Histone H3 on pSer10, indicating mitotic cells with condensed DNA, and cyclin-dependent kinase 2 (Cdk2) on pTyr15, indicating that cells are at the G1/S transition, were analyzed by using the Cell Cycle In-Cell ELISA kit (#ab140363, Abcam, Cambridge, UK) assay that employs quantitative immunocytochemistry. Briefly, melanoma cells (10^4^/well) were seeded onto 96-well plates and incubated with 20 µM OA or its vehicle (Ctrl). After 72 h, cells were fixed with paraformaldehyde 4% and, then, incubated with 0.02% sodium azide to decrease background signal. Following the manufacturer’s instructions, in each well, the Cdk2-pTyr15 and Histone H3-pSer10 levels were detected by the incubation of specific primary and, then, enzyme-linked secondary antibodies conjugated to horseradish peroxidase or alkaline phosphatase which generate signal through two spectrally distinct fluorogenic substrates. Each signal was normalized to the total cell amount of corresponding well by using Janus Green stain (normalized intensity).

### Internucleosomal DNA Fragmentation

Apoptosis in 501Mel cells treated with 20 µM OA for 72h was assessed by using Cell Death Detection ELISA plus (#11774425001, Sigma-Aldrich, Milan, Italy), as previously reported by Carpi and colleagues (Carpi et al., 2018).

### Gene Expression Analyses

Total RNA from cells was extracted using the RNeasy Mini kit. Reverse-transcription and Real-time PCR were carried out one-step by using the QuantiNova SYBR Green RT-PCR Kit (Qiagen, Hilden, Germany) on 200 ng of each sample, following the manufacturer’s instructions. The sequences of forward and reverse primers are reported in **Table 3**. Signals were detected on the MiniOpticon CFX 48 real-time PCR Detection System (Bio-Rad, Hercules, CA, USA). The mRNA expression was calculated using the 2^-δδCt^ method.

**Table 3:** Nucleotide sequences of primers used for real-time PCR.

Gene	RefSeq	Primer sequence (5′—3′)	Size (bp)
BCL2	NM_000633.3	Forward TCCATGTCTTTGGACAACCA Reverse CTCCACCAGTGTTCCCATCT	20 20
BAX	NM_001291429.2 NM_004324.4	Forward TCTGACGGCAACTTCAACTG Reverse TTGAGGAGTCTCACCCAACC	20 20
MCL-1	NM_021960.5	Forward CCAAGAAAGCTGCATCGAACCAT Reverse CAGCACATTCCTGATGCCACCT	23 22
K-RAS	NM_033360.4 NM_001369786.1	Forward CAGTAGACACAAAACAGGCTCAG Reverse TGTCGGATCTCCCTCACCAATG	23 22
c-KIT	NM_001385286.1 NM_001385292.1 NM_001385288.1 NM_001093772.2 NM_001385284.1 NM_001385285.1 NM_001385290.1 NM_000222.3	Forward GGATCACGGAAAAGGCAGAA Reverse GGCAGGATCTCTAACAAACACATAAA	20 26
PIK3R3	NM_001303427.2 NM_001328653.2 NM_001328654.2 NM_001303429.2 NM_003629.4 NM_001328651.1 NM_001328650.1 NM_001328649.1 NM_001328648.1 NM_001303428.1 NM_001114172.1	Forward CTTGCTCTGTGGTGGCCGAT Reverse GACGTTGAGGGAGTCGTTGT	20 20
mTOR	NM_004958.4 XM_024446187.1 XM_017000902.1 XM_011541166.2 XM_017000901.1 XM_005263438.2	Forward ATGCAGCTGTCCTGGTTCTC Reverse AATCAGACAGGCACGAAGGG	20 20
β-actin	NM_001101.5	Forward GTCATTCCAAATATGAGATGCGT Reverse GCATTACATAATTTACACGAAAGCA	23 25

### MicroRNA Expression Analyses

Among tumor-related miRNAs, using bioinformatic tools (TargetScanHuman, version 7.2 (Agarwal et al., 2015) and miRTargetLink in Human (Hamberg et al., 2016)) we selected for analysis miRNAs showing an experimentally validated direct interaction with the OA deregulated transcripts in melanoma cells. In detail, hsa-miR-214-3p targets BAX, hsa-miR-34a-5p targets BCL2 and c-KIT, hsa-miR-16-5p targets BCL2, K-RAS and MTOR, hsa-miR-193a-3p targets MCL-1, c-KIT and K-RAS, hsa-miR-155-5p targets K-RAS and hsa-miR-193a-5p targets PIK3R3 and MTOR.

The miRNeasy Mini Kit (Qiagen, Hilden, Germany) was used for purification and extraction of total miRNAs. The extracted miRNAs were retro-transcribed by the miScript Reverse Transcription Kit (Qiagen, Hilden, Germany) and the corresponding cDNA was diluted 1:10 in RNase-free water. The miScript SYBR-Green PCR kit (Qiagen, Germany) was used to perform qPCR experiments in triplicate. Signals were detected on the MiniOpticon CFX 48 real-time PCR Detection System (Bio-Rad, Hercules, CA, USA). MiScript Primer Assays specific for hsa-miR-214-3p (MIMAT0000271), hsa-miR-34a-5p (MIMAT0000255), hsa-miR-16-5p (MIMAT0000069), hsa-miR-193a-3p (MIMAT0000459), hsa-miR-155-5p (MIMAT0000646), hsa-miR-193a-5p (MIMAT0004614), and hsa-SNORD6 were obtained from Qiagen. The miRNA expression was calculated using the 2^-δδCt^ method and the SNORD6 gene was used as housekeeping.

### Statistical Analysis

Data were presented as mean ± standard deviation (SD) of at least three independent experiments. All statistical procedures were performed by commercial software (GraphPad Prism, version 8.0 from GraphPad Software Inc., San Diego, CA, USA). Student’s t-test for unpaired data was performed to compare two groups. A p value < 0.05 was considered statistically significant.

## Results

### OA Inhibits Melanoma Cell Viability

**Figure 1:**
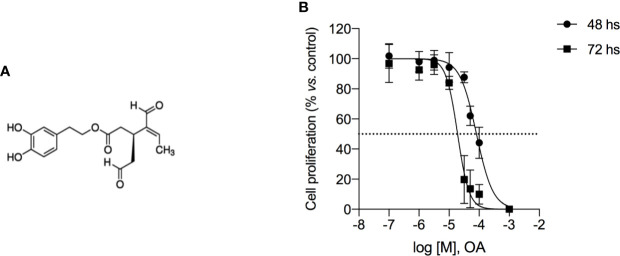
Oleacein inhibits the proliferation of human melanoma cells 501Mel. **(A)** Chemical structure of oleacein. **(B)** 501Mel cells were treated with increasing concentration (0.1–200 μM) of oleacein. Growth inhibition was measured at 48 and 72 h using the MTT assay and is expressed as percentage of Ctrl (vehicle-treated cells). Data are presented as means ± SD of three independent experiments, each performed in triplicate.

OA (chemical structure reported in **Figure 1A**) induced cell growth inhibition in 501Mel cells, showing an IC50 mean value of 81.9 ± 6.9 µM and 19.1 ± 5.8 µM after 48 and 72 h of treatment, respectively (**Figure 1B**). This ability to reduce cell viability was time- and concentration-dependent and then the maximum tested concentration of OA (200 µM) showed to completely inhibit cell proliferation at both time points.

### OA Induces G1 Cell Cycle Arrest

To investigate the mechanism underlying the inhibition of melanoma cell viability by OA, the analysis of the cell cycle profile of OA-treated cells was investigated by the evaluation of Histone H3-pSer10, a marker of mitosis, and Cdk2-pTyr15, a marker of the G1/S transition.

**Figure 2:**
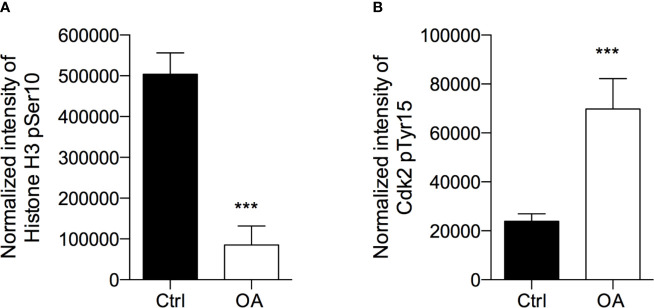
Oleacein induces a cell cycle arrest in G1/S phase transition. 501Mel cells were treated with OA 20 µM for 72 h. Phosphorylation levels of Histone H3 at pSer10 **(A)** and Cdk2 at pTyr15 **(B)** were expressed as fluorescence unit normalized on the corresponding cell amount (Normalized intensity). Data are presented as means ± SD of three independent experiments, each performed in triplicate. Student-t test was performed; ***p < 0.001 compared to the corresponding control (vehicle-treated cells, Ctrl).

After treatment with OA at the IC50 concentration for 72 h, we observed that H3-pSer10 levels were significantly decreased (5.9 fold-change) compared to control cells (**Figure 2A**). The phosphorylation of H3 on Ser10 occurs in late G2 phase and proceeds during mitosis up to prophase when chromosomes condense; at the end of mitosis, H3 is totally dephosphorylated (Prigent, 2003). Therefore, the significant reduction of this phosphorylation indicates that OA treatment did not induce a cell cycle block in G2/M transition.

In parallel, in OA treated cells we observed a significant increase (2.9 fold-increase) of the phosphorylation of Cdk2 on Tyr15 (**Figure 2B**), consistent with a cell cycle arrest in G1/S transition. Indeed, Cdk2 is a master regulator of G1/S transition (Welburn et al., 2007) which triggers out when it is in the dephosphorylated active form; the phosphorylation on Tyr15 leads to inactivation of this cyclin. Therefore, the high phosphorylation observed in melanoma cells after OA treatment suggested a block of melanoma cells in G1 transition.

### OA Induces Apoptosis

To better understand the mechanism behind the cell growth inhibition of OA, its role in apoptosis induction was evaluated. To this aim, the analysis of internucleosomal DNA fragmentation, a phenomenon that occurs during late stages of programmed cell death, and the evaluation of expression levels of genes and miRNAs involved in the apoptotic process were performed.

**Figure 3:**
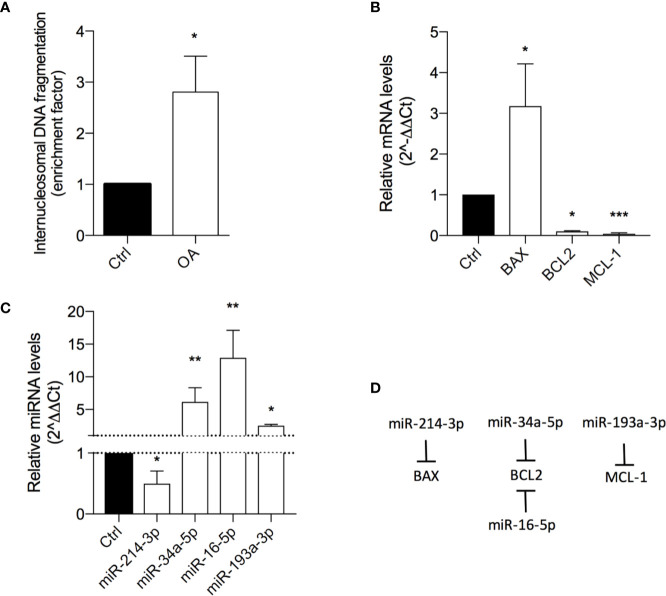
Oleacein induces apoptosis in 501Mel cells. **(A)** Internucleosomal DNA fragmentation in cells treated with 20 μM oleacein for 72 h, compared to control (vehicle-treated) cells. Expression levels of mRNAs **(B)** and microRNAs (miRNAs) **(C)** involved in the apoptosis regulation in cells treated with 20 µM oleacein for 72 h, expressed as fold over control. **(D)** Schematic representation of the miRNA-mRNA targeting. Data are presented as means ± SD of three independent experiments, each performed in triplicate. Student-t test was performed. *p < 0.05, **p < 0.01, ***p < 0.001, compared to the corresponding control.

The result showed OA inducing a 3-fold increase of DNA histone fragments into cell cytoplasm, revealing a proapoptotic effect (**Figure 3A**).

The ability of OA to induce apoptosis in treated cells was confirmed by the analysis of transcriptional expression levels of genes and miRNAs involved in apoptosis regulation. Indeed, we observed the modulation of mRNA levels of the Bcl-2 family core members BAX, BCL2, and MCL-1 (**Figure 3B**; **Table S1**). In detail, the 72h exposure to OA (at the IC50 concentration) induced a strong increase of the proapoptotic BAX mRNA levels (3-fold-change) and a marked decrease of the antiapoptotic BCL2 (10 fold-change) and MCL-1 (24 fold-change) transcriptional levels.

To further evaluate the modulatory effect of OA on apoptosis regulation, the expression of some miRNAs, short non-coding RNAs able to post-transcriptionally regulate gene expression, was evaluated. In detail, miRNAs regulating the expression of apoptotic genes previously observed as deregulated by OA were selected (**Figure 3D**). Concordantly with gene modulation results, a significant 2-fold decrease of miR-214-3p, targeting BAX, and a significant upregulation of miR-34a-5p and miR-16-5p, both targeting BCL2, and of miR-193a-3p, targeting MCL-1 (**Figure 3C**) were observed.

### OA Inhibits mTOR Pathway

One of the most important pathways involved in the regulation of cell proliferation, survival and apoptosis is mTOR pathway (Sabatini, 2006; Karbowniczek et al., 2008). Consequently, the potential effect of OA on the expression of genes and miRNAs linked to this signaling was investigated.

**Figure 4:**
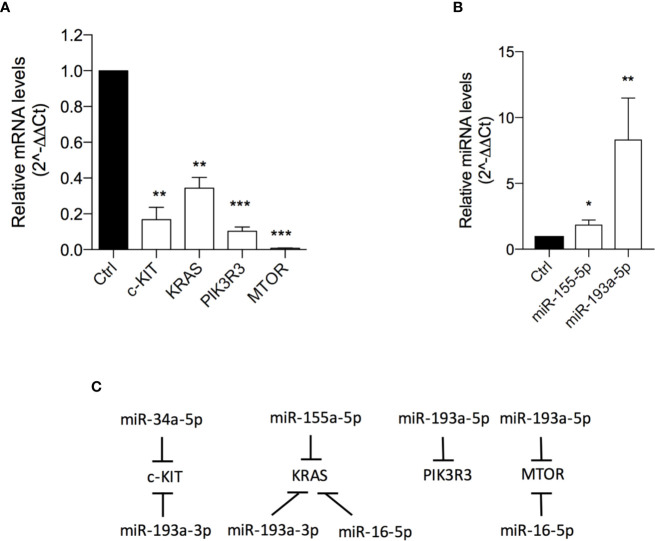
Oleacein downregulates genes and miRNAs involved in mammalian target of rapamycin (mTOR) pathway. Expression levels of genes **(A)** and microRNAs (miRNAs) **(B)** related to mTOR pathway in cells treated with 20 µM oleacein for 72 h. **(C)** Schematic representation of the miRNA-mRNA targeting. Data are presented as means ± SD of three independent experiments, each performed in triplicate. Student-t test was performed, *p < 0.05; **p < 0.01; ***p < 0.001 compared to the corresponding control.

501Mel cells exposed to OA (at the IC50 concentration for 72h) showed a significant down-expression of C-KIT (6 fold-change), KRAS (3 fold-change) and PIK3R3 (9.8 fold-change) and, interestingly, an almost total reset of the mTOR transcriptional levels (**Figure 4A**; **Table S2**). In agreement with these data, a significant upregulation of miR-155-5p (targeting KRAS and PIK3R3) and miR-193a-5p (targeting mTOR) was evident after OA treatment (**Figures 4B, C**).

The observed transcriptional downregulation of C-KIT, K-RAS and mTOR is also in agreement with the upregulation of some miRNAs already described in **Figure 3**. Indeed, C-KIT is also targeted by miR-34a-5p and miR-193a-3p, K-RAS is also a target of miR-193a-3p and miR-16-5p, and the expression of mTOR is further regulated by miR-16-5p (**Figure 4C**).

### Oleacin^®^ and Imiquimod Comparably Decrease Melanoma Cell Viability

**Figure 5:**
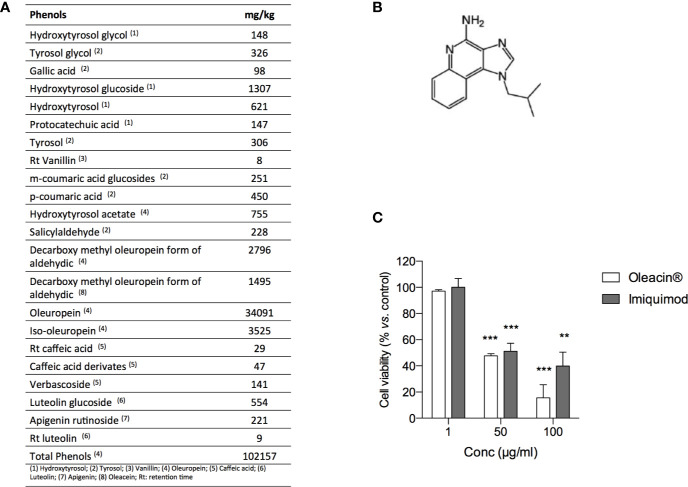
Oleacin^®^ and Imiquimod decrease 501Mel cell viability. **(A)** Polyphenol composition of Oleacin^®^. **(B)** Chemical structure of Imiquimod. **(C)** 501Mel cells were treated with 1, 50, and 100 µg/ml of either Oleacin^®^ or Imiquimod. Growth inhibition was measured at 72 h using the MTS assay and is expressed as percentage of Ctrl (corresponding vehicle-treated cells). Data are presented as means ± SD of three independent experiments, each performed in triplicate. Student-t test was performed, **p < 0.01; ***p < 0.001 compared to the corresponding control.

In order to investigate the antimelanoma activity of a natural formulation rich in OA for potential topic application, we selected the glycerol concentrated extract of olive leaf polyphenols commercially named “Oleacin^®^”. The composition of Oleacin^®^ showed the presence of a great amount of polyphenols (102157 mg/kg) including OA and its derivatives, as reported in **Figure 5A**.

The ability of Oleacin^®^ to modulate melanoma cell proliferation was compared to that of Imiquimod (**Figure 5B**), reference drug used topically as first- or second- line treatment of melanoma *in situ* (National Comprehensive Cancer Network®, 2020).

For this purpose, 501Mel cells were treated with Oleacin^®^ or Imiquimod at three different concentrations (1, 50, and 100 µg/ml) for 72h. We here selected only three concentrations in order to provide preliminary evidence of efficacy, thus paving the way for further studies on Oleacin^®^ alone and in combination. As reported in **Figure 5C**, either Oleacin^®^ or Imiquimod inhibited melanoma cell growth in a concentration-dependent manner. In detail, we found that neither Oleacin^®^ nor Imiquimod was able to modulate cell growth at 1 µg/ml, the lower dose tested, while both significantly decreased 501Mel cell viability at 50 and 100 µg/ml in a comparable manner, compared to the corresponding control. Interestingly, the concentration of OA is 0.23 µM in 50 µg/ml of Oleacin^®^ and 0.47 µM in 100 µg/ml of Oleacin^®^, i.e. about 100-fold and 50-fold lower compared to the IC50 of OA alone (≈20 µM).

## Discussion

Growing evidence have corroborated the chemopreventive role of secoiridoids derived from *Olea Europeae* L. olive and EVOO, such as Oleuropein, OA, Oleocanthal, and others (Fabiani, 2016; Castejón et al., 2020). Although a great deal of research was devoted to characterizing the anticancer effects of these polyphenols, the effect of OA in tumor models remains poorly studied (Fabiani et al., 2008; Polini et al., 2018; Juli et al., 2019; Cirmi et al., 2020).

**Figure 6:**
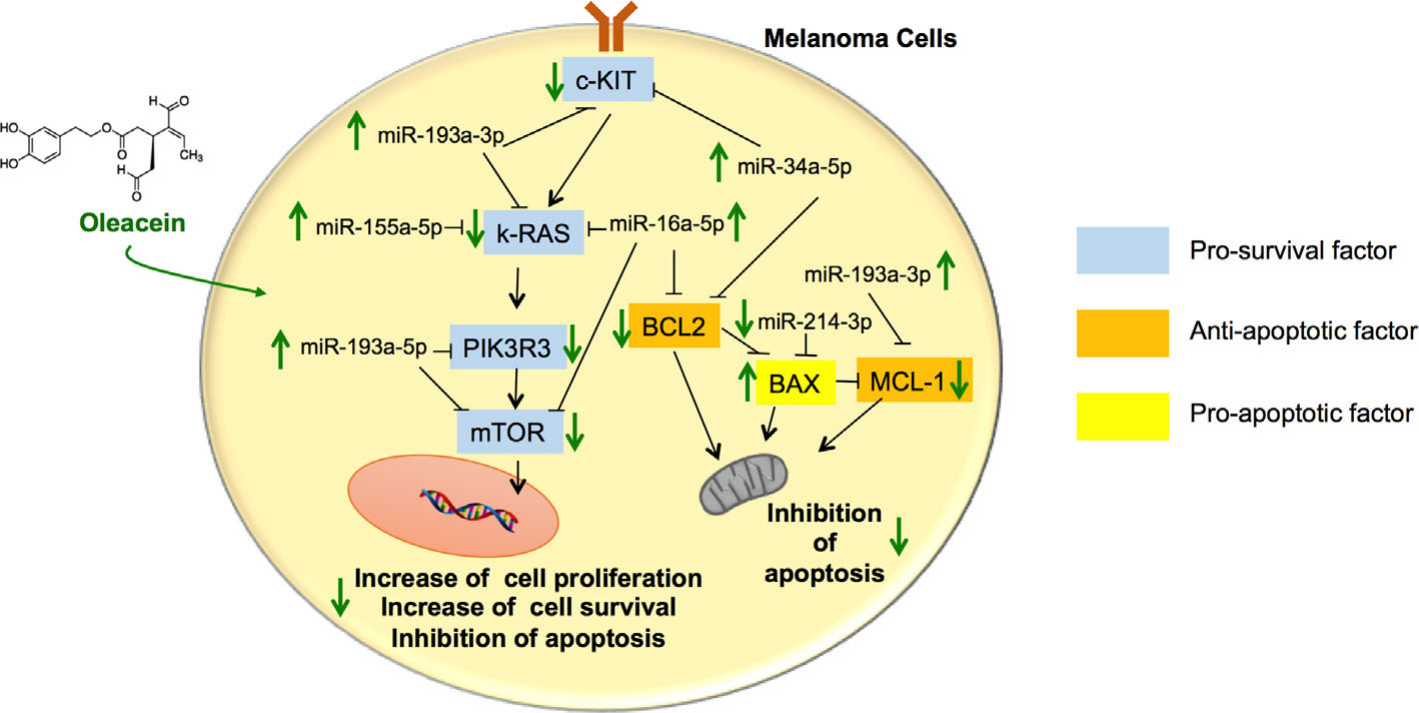
Schematic summary of the pathways involved in the tumor suppressor activity of oleacein in melanoma cells. Oleacein regulates the expression pattern of genes and miRNAs so that to inhibit proliferation, survival and to induce apoptosis of melanoma cells. See text for further details. The black arrows indicate stimulation. The black lines indicate inhibition.

As far as we know, this is the first time that the activity of OA in melanoma cells has been addressed. Our results show that OA exerts antimelanoma activity by targeting different genes and their epigenetic actors linked to survival and apoptosis, including the Bcl-2 family and mTOR pathway, leading to inhibition of survival and induction of apoptosis (see schematic picture in **Figure 6**).

A hallmark of most cancers, including melanoma, is an imbalanced expression of pro- and antiapoptotic agents of Bcl-2 family (Soengas and Lowe, 2003). The Bcl-2 family governs the fate of cells through the control of the intrinsic pathway of apoptosis. The main mechanism of action of the Bcl-2 family is the regulation of cytochrome C release from the mitochondria *via* alteration of mitochondrial membrane permeability (Elmore, 2007). In detail, some members of the Bcl-2 family, BCL-2 and MCL1, inhibit the release of cytochrome C while others, such as BAX, increase cytochrome C release from the mitochondria, thus inhibiting and activating the apoptotic machine, respectively (Elmore, 2007).

Through the interference with the intrinsic pathway of apoptosis, these proteins are implicated in melanoma cell survival, proliferation, and chemo-resistance (Lee et al., 2019) (McKee et al., 2013). Thus, controlling the expression of pro- and antiapoptotic mediators represents a highly potent stimulus to trigger apoptosis in melanoma cells.

Interestingly, we demonstrated that OA promotes cell growth arrest by inducing apoptosis in melanoma cells through the inhibition of BLC2 family pathway. Indeed, in OA-treated cells, we reported the decrease of the antiapoptotic BCL-2 and MCL1 mRNA expression and the increase of their experimentally validated inhibitory miRNAs, i.e., miR-34a-5p, miR-16-5p, and miR-193a-3p, (miRTarBase Accession ID: MIRT002298, MIRT001800, and MIRT002485) and the simultaneous increase of proapoptotic BAX expression and decrease of its silencing miR-214-3p (miRTarBase Accession ID: MIRT438124). These data indicate that OA exerts its cytotoxicity activity against melanoma cells controlling, at the epigenetic level, cell machinery through changes of miRNAs expression and their downstream mRNA targets. A similar modulatory effect by OA on BCL-2 and BAX has been observed in neuroblastoma cells (Cirmi et al., 2020). The ability of OA to exert epigenetic modulation has been also demonstrated in other pathophysiological models, including adipocyte inflammation for miR-155 and miR-34a (Carpi et al., 2019) and multiple myeloma for miR-29b and miR-22 with the downregulation of several class I/II histone deacetylases (Juli et al., 2019; Cuyàs et al., 2019).

Furthermore, we observed that the treatment with OA led to significant modulation of mTOR pathway. mTOR is a kinase that integrates growth factor stimulation and nutrient availability with cell growth and protein translation (Sabatini, 2006). This pathway is frequently found constitutively activated in melanoma (Karbowniczek et al., 2008), and thus is considered a growth-promoting factor in melanoma. Interestingly, high levels of mTOR expression can lead to apoptotic resistance by modulating several molecules, including Bcl-2 family members, and thus promoting tumor cell survival (Castedo et al., 2002; Asnaghi et al., 2004)). The activated PI3K/Akt/mTOR pathway has been shown to induce upregulation of antiapoptotic Bcl-2 family member, such as MCL1 (Rahmani et al., 2013; McKee et al., 2013), and the phosphorylation of BAX at the S184 site leading to BAX inactivation (Wang et al., 2010; Li et al., 2017).

Our findings show that OA concordantly decreases the mRNA expression of c-KIT, K-RAS, and PIK3R3, important effectors responsible for intensified mTOR activation. OA counteracts the expression of these genes by inducing an increase in the expression of miR-34a-5p, miR-193a-3p, miR-193a-5p, miR-16-5p, and miR-155-5p, which act as regulatory elements able to decrease transcriptional levels of these target genes (c-KIT, K-RAS, and PIK3R3) (miRTarBase Accession ID: MIRT438239, MIRT005100, MIRT502085, MIRT031485) (Gao et al., 2011; Yu et al., 2015; You et al., 2016), and hence contrasting mTOR signaling. Moreover, mTOR expression levels were downregulated also through the increased expression of its silencing agents, miR-16-5p and miR-193a-5p, in OA-treated melanoma cells. These data indicate, for the first time, the ability of OA to interfere with the activation of mTOR pathway at the transcriptional level of its main effector proteins, by modulating the expression of the corresponding miRNAs. This miRNA-modulating activity of OA is shared with other polyphenols (Milenkovic et al., 2013) and represents another mode of action of polyphenols at the molecular level underlying their beneficial effects in different pathophysiological contexts, including cancer (Pandima Devi et al., 2017). miRNAs silence the gene functions by targeting mRNA through degradation or translation repression. The role for miRNAs in the molecular pathogenesis of cancer has clearly emerged with the demonstration of their deregulation in several types of cancers and their involvement in both oncogenic and tumor suppressor pathways, regulating cell proliferation, differentiation and apoptosis (Pandima Devi et al., 2017). Therefore, targeting cancer-associated miRNAs might provide novel and alternative treatment strategy in cancer therapy. Further studies using specific antago-miRNAs will be performed to substantiate the causal involvement of specific miRNAs in the anticancer mechanism of action of OA.

Despite great interest in mTOR inhibitors in cancer treatment, a clinical study conducted in patients with metastatic unresectable malignant melanoma treated with everolimus (mTOR inhibitor) has shown limited effects (Dronca et al., 2014). The authors suggested that other mechanisms in the biology of melanoma allow mTOR pathway to be bypassed (Dronca et al., 2014), and one of the suggested mechanisms involves molecules controlling apoptosis. Indeed, data obtained in other solid tumors showed that inhibition of both mTOR signaling and Bcl-2 family activity did trigger *in vivo* tumor regressions (Preuss et al., 2013; Jebahi et al., 2014; Iacovelli et al., 2015; Li et al., 2017). Therefore, OA possesses the ability of a dual control in melanoma, i.e. antiapoptosis and mTOR inhibition, revealing an interesting potentiality as a novel antitumor drug scaffold. The here demonstrated direct antigrowth action of OA in melanoma cells significantly occurs at a low micromole concentrations that are instead not toxic in different non-cancerous cells (Czerwińska et al., 2014; Filipek et al., 2015; Carpi et al., 2019), suggesting a specific targeting for mechanisms hyper-reactive in cancer, and could be complemented by antiinflammatory and antioxidant effects of OA (Lozano-Castellón et al., 2019; Carpi et al., 2019) in protecting against cancer.

Natural product and natural molecules played and continue to play a highly relevant role in the process of drug discovery and development (Newman and Cragg, 2015). Among the small antitumor drugs, approved between 1930 and 2012, the 67% was inspired by or derived from natural products (Newman and Giddings, 2014). In this context, experimental, epidemiological, and clinical associations between the consumption of some foods and beverages containing phytochemicals, such as phenols and polyphenols of *Olea Europeae* L. tree, and the onset of several types of cancers, including cutaneous melanoma, establishes an interesting starting point for the discovery of novel drugs, and in this context OA appears to hold promise.

In addition to the fruit (from which EVOO is derived), the leaves of the olive plant also contain phenolic compounds at a concentration and combination different from the olive fruit and oil (Kountouri et al., 2007). Increasing evidence have demonstrated relevant healthful protective properties of leaves of the olive plant in different pathophysiological contexts including cancer and cardiovascular diseases (Lockyer et al., 2012; Boss et al., 2016). Based on our results with OA, we performed a preliminary evaluation of the direct antimelanoma activity of a glycerol concentrated extract of leaves of the olive plant, which is a mixture mostly containing simple phenols including hydroxytyrosol and its secoiridoid derivatives oleuropein and OA. The results indicate a significant inhibition of melanoma cell vitality by the extract similar to that of Imiquimod, thus laying the basic foundation for following investigations for potential topic application against cutaneous melanoma. Interestingly, the antimelanoma efficacy of the extract may depend on the highly content of polyphenols for which this ability has been previously reported, as for hydroxytyrosol and oleuropein (D’Angelo et al., 2005; Ruzzolini et al., 2018), or here demonstrated for the first time, as for OA. Of course, the mechanisms of action of different polyphenols might be different, and a multi-targeted antitumor activity could be inferred. It is experimentally demonstrated that the health effects of polyphenol-rich plants, foods and beverages as well as their extracts, frequently used as nutraceuticals, are contributed by the synergistic/additive action of structurally different compounds present in complex mixture, that may improve the capacity to enhance health outcomes. We cannot exclude that such synergic interaction among different polyphenols occurs for Oleacein^®^. We indeed noticed that the lowest concentration of Oleacein^®^ effective in inhibiting cell viability (50 µg/ml) corresponds to an OA concentration of 0.23 µM, 100-fold lower compared to the IC50 of OA alone (≈20 µM). However, these observations warrant further mechanistic studies as well as clinical trials to substantiate the potential anticancer potential of Oleacein^®^ as well as its components.

The present study has several limitations. First, from the data here presented we could infer the involvement of the studied miRNAs in the proapoptotic and antimelanoma activity of OA, because our data only provided associations between miRNA modulation and antitumor activity by OA. A causal role of these miRNAs in the action of OA should be more directly assessed by using specific antagomiRs. Second, the OA-induced G1 arrest of melanoma cells should be confirmed by flow cytometry experiments, and similarly other apoptosis assays such as caspase activation, TUNEL staining, and flow cytometry, might substantiate the ability of OA to induce apoptosis in melanoma cells. Finally, experiments combining OA with Imiquimod as well as other chemotherapeutic drugs might evaluate whether OA has the ability to sensitize tumor cells to drug effects, thus potentiating current chemotherapies. All these limitations will be the object of future investigations.

In conclusion, our study is the first demonstration of an antitumor activity of OA by controlling the altered pattern of gene expression (transcripts and miRNAs) related to mTOR and BCL2 pathways in melanoma cells. The precise mode of action of OA and the functional implications remain to be determined, but our *in vitro* results provide novel basic knowledge about possible mechanisms underlying the antitumor benefit ascribed to polyphenols of *Olea Europeae* L. tree. A more thorough and accurate exploration at the protein level of the OA modes of action and its combination with antimelanoma treatments will be the target of next studies. Furthermore studies are warranted to recapitulate these findings in animal models in preventing the onset of primary melanoma or/and antagonizing the evolution of melanoma.

## Data Availability Statement

The raw data supporting the conclusions of this article will be made available by the authors, without undue reservation, to any qualified researcher.

## Author Contributions

SC, BP, ES, and PN participated in research design. SC, BP, MD, and JS conducted the experiments. CM, MD, JS, and MM contributed new reagents or analytic tools. SC, BP, JS, MD, and CM performed the data analysis. SC, BP, ES, and PN wrote or contributed to the writing of the manuscript.

## Funding

The study was in part supported by Arisi S.s. Società Agricola, which had no role in study design, collection, analysis, and interpretation of data, in the writing of the manuscript and in the decision to submit the paper for publication. The study was partially supported by University of Pisa funds of SC and PN and Associazione contro il melanoma (ACM OdV).

## Conflict of Interest

The authors declare that the research was conducted in the absence of any commercial or financial relationships that could be construed as a potential conflict of interest.
