# Supplementary material for: miRNA Modulation and Antitumor Activity by the Extra-Virgin Olive Oil Polyphenol Oleacein in Human Melanoma Cells
Source: Front Pharmacol. 2020 Sep 23;11:574317. doi: 10.3389/fphar.2020.574317 (PMC7539365; doi:10.3389/fphar.2020.574317)
Supplement: Supplementary file 1 [file DataSheet_1.docx]

**Supporting Information**

**Table S1**. The dataset presented in Figure 3B.

| Ctrl | BAX | BCL2 | MCL-1 |
| --- | --- | --- | --- |
| 1,00 | 2,713 | 0,119 | 0.076 |
|  | 4,362 | 0,101 | 0.000 |
|  | 2,4452 | 0,080 | 0.048 |

**Table S2.** The dataset presented in Figure 4A.

| Ctrl | c-KIT | KRAS | PIK3R3 | MTOR |
| --- | --- | --- | --- | --- |
| 1 | 0,142 | 0,323 | 0,073 | 0,0103 |
|  | 0,062 | 0,455 | 0,149 | 0,008 |
|  | 0,296 | 0,253 | 0,083 | 0,004 |
